# Converging Signaling Networks Drive Taste Bud Morphogenesis, Turnover, and Regeneration

**DOI:** 10.3390/ijms27135644

**Published:** 2026-06-23

**Authors:** In Young Jo, Jin-Woo Kim, Jae Kyeom Kim, Jeong-Oh Shin

**Affiliations:** 1 Department of Radiation Oncology, Soonchunhyang University Cheonan Hospital, Cheonan 31151, Republic of Korea; 2 Department of Oral and Maxillofacial Surgery, Research Institute for Intractable Osteonecrosis of the Jaw, College of Medicine, Ewha Womans University, Seoul 07804, Republic of Korea; 3 Department of Food Biotechnology, Korea University, Sejong 30019, Republic of Korea; 4 Department of Health Behavior and Nutrition Sciences, University of Delaware, Newark, DE 19711, USA; 5 Department of Anatomy, College of Medicine, Kangwon National University, Chuncheon 24341, Republic of Korea

**Keywords:** taste bud, WNT/β-catenin, SHH, SOX2, LGR5, R-spondin, POU2F3, organoid, regeneration, lineage specification

## Abstract

Buds are continuously renewed sensory organs in which development, adult maintenance, and repair share overlapping molecular circuitry. During embryogenesis, WNT/β-catenin signaling promotes taste placode formation and placodal *Shh* expression, while SHH refines papilla spacing and restricts neighboring papilla formation. SOX2 functions as a taste-competence and progenitor maintenance factor. In adults, LGR5/LGR6–RSPO–WNT signaling sustains progenitor activity, and gustatory neurons are an important source of RSPO2; available genetic evidence is consistent with a neuron-derived contribution to the LGR5/LGR6 niche, and AAV-Cre-mediated neuron-specific ablation of *Rspo2* in the petrosal ganglion led to near-complete loss of circumvallate taste buds. HH signaling from epithelial and neuronal sources further supports SOX2-dependent progenitor homeostasis. Lineage allocation is governed by transcriptional programs that include POU2F3/SKN-1a for sweet, umami, and bitter type II taste receptor cells, and ASCL1 with posterior-field NKX2-2 for type III presynaptic/sour cells. After denervation or irradiation, regeneration depends primarily on LGR5^+^/KRT14^+^ progenitors and may be supplemented, in specific injury contexts, by plasticity of a subset of K8-lineage taste receptor cells that acquire KRT14/SOX2/PCNA progenitor-like features. Key unresolved questions include the direct chromatin targets of taste lineage regulators (which remain to be defined by ChIP-seq in native taste progenitors), the identity of the type I cell selector, the contribution of dedifferentiation across injury models, and the degree to which mouse-derived networks are conserved in human taste biology.

## 1. Introduction

Taste allows vertebrates to evaluate the nutritional value and potential toxicity of ingested material. This sensory task is performed by taste buds, compact epithelial sensory organs composed of approximately 50–100 cells embedded in the epithelium of the tongue, palate, pharynx, and larynx [1,2]. A defining feature of taste buds is continuous cellular turnover of mature taste receptor cells from a self-renewing progenitor pool. In adult mice, differentiated taste receptor cells (TRCs) are replaced over a period of roughly 8–12 days in fungiform papillae and up to 22 days in circumvallate papillae, requiring persistent progenitor activity and lineage allocation throughout life [3]. On the mouse tongue, taste buds reside in three major papilla types. Fungiform papillae (FuP) occupy the anterior tongue and generally contain a single taste bud. The circumvallate papilla (CVP) is posterior midline and harbors hundreds of taste buds. Foliate papillae (FoP) are scattered along the posterior-lateral tongue margins [4]. FuP taste buds receive their innervation mainly from the chorda tympani branch of the facial nerve (CN VII). CVP taste buds are innervated by the glossopharyngeal nerve (CN IX). Foliate papillae taste buds are dually innervated by both CN VII and CN IX. This innervation is required not only for sensory transmission but also for ongoing maintenance of the epithelial taste organ. Genetic and organoid studies have shown that pathways used during taste organ development, including WNT/β-catenin, SHH/HH, SOX2, *Notch*, and BMP signaling, are redeployed during adult maintenance but with altered functional outputs [3,5]. The identification of gustatory-neuron-associated R-spondins, particularly RSPO2, as candidate WNT-amplifying niche signals has provided a molecular framework for the long-recognized dependence of taste buds on gustatory innervation [6]. An additional layer of organization arises from the composite embryonic origin of the tongue. Lineage tracing studies indicate that anterior tongue epithelium, including FuP, is predominantly ectoderm-derived, whereas posterior CVP/FoP fields are endoderm-derived [7]. This anterior–posterior distinction affects transcription factor usage, progenitor composition, and the interpretation of genetic perturbation experiments [8]. Taste bud biology therefore requires region-specific models rather than a single uniform program.

This review integrates evidence for the converging signaling network that governs taste bud development, homeostasis, and regeneration. The discussion proceeds from embryonic placode patterning through adult progenitor maintenance, lineage specification, injury responses, organoid systems, and comparative vertebrate models. Throughout, we distinguish established mechanisms from plausible but untested regulatory models, and we address signaling roles separately for embryonic patterning, adult homeostasis, and regeneration, and separately for anterior (ectoderm-derived) and posterior (endoderm-derived) fields. Terms such as ‘primary axis’ and ‘network’ are used descriptively rather than as claims of mechanistic primacy. Our synthesis overlaps in scope with two prior reviews—Piarowski, Isner and Barlow [9] and Barlow (WIREs Mech Dis, 2022)—to which we refer the reader for complementary coverage. The present contribution is distinguished by two features: (i) the integration of work published in 2025–2026—including the tripotency of posterior tongue stem cells, the genetic dissection of *Rspo2*, the role of c-*Kit*/KIT in taste cell resilience, anterior tongue organoids, and the single-cell atlas of taste papilla aging—within a single network framework; and (ii) a consistent emphasis on the ectoderm–endoderm regional division of the tongue and on chromatin-level experiments (e.g., ChIP-seq in native taste progenitors) that the field has not yet performed.

## 2. Anatomical and Cellular Framework

### 2.1. Taste Fields and Their Embryonic Origins

**Figure 1:**
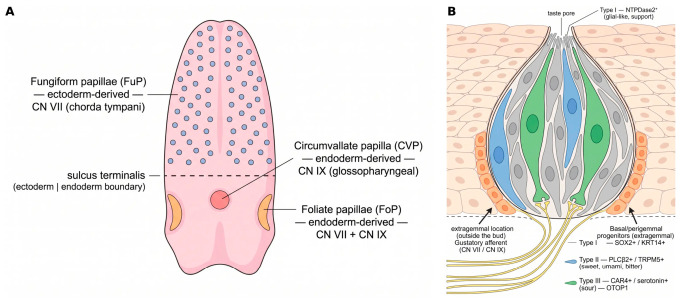
Anatomy of the mouse tongue and the mature taste bud. (**A**) Dorsal view of the mouse tongue showing the distribution and developmental origins of taste papillae. Fungiform papillae (FuP, blue dots) are located in the anterior two-thirds, are ectoderm-derived, and receive innervation from the chorda tympani branch of the facial nerve (CN VII). The circumvallate papilla (CVP) lies at the posterior midline, is endoderm-derived, and is innervated by the glossopharyngeal nerve (CN IX). Foliate papillae (FoP) are located along the posterior-lateral margins, are endoderm-derived, and receive dual innervation from both CN VII and CN IX. The dashed line indicates the approximate sulcus terminalis boundary between ectoderm- and endoderm-derived territories. (**B**) Cross-section of a mature taste bud illustrating the principal cell populations: type I glial-like support cells (gray; NTPDase2^+^), type II receptor cells (blue; PLCβ2^+^/TRPM5^+^; sweet, umami, and bitter), type III presynaptic cells (green; CAR4^+^/serotonin^+^; sour), and basal/perigemmal progenitor cells (orange; SOX2^+^/KRT14^+^; extragemmal location). Gustatory afferent nerve fibers (CN VII/CN IX) innervate the taste bud from below.

A central principle in taste biology is that the major tongue papilla types do not share a single germ-layer origin (Figure 1). In mice, inducible Cre-based lineage tracing by Rothova et al. demonstrated that the anterior tongue epithelium giving rise to FuP is predominantly ectoderm-derived, whereas the posterior tongue epithelium forming CVP and FoP is endoderm-derived [7]. This anterior-ectoderm/posterior-endoderm organization has implications for transcription factor expression, progenitor identity, and local signaling during taste bud maintenance [8]. The approximate boundary between these territories lies near the foramen cecum/posterior tongue landmark region rather than functioning as a sharply demarcated mature anatomical line in the mouse [4]. The anterior field shows broad SOX2-associated epithelial competence and gives rise to FuP, each usually containing a single taste bud innervated by CN VII. The posterior-field forms the CVP and FoP, which contain numerous taste buds and are densely innervated by CN IX [1].

This dual-field organization is important experimentally. Perturbations targeted to one papilla type cannot be assumed to generalize across the entire gustatory system, because phenotypes frequently differ between anterior FuP and posterior CVP/FoP taste fields, and because the underlying transcription factor codes—for example, the posterior-restricted requirement for NKX2-2 in type III lineage [8]—are themselves region-specific.

### 2.2. Cellular Organization of the Mature Taste Bud

Within the mature taste bud, several cell populations can be distinguished by morphology, molecular markers, and function (Figure 1). Type I cells are glial-like support cells that wrap other cell types and express the ectoenzyme NTPDase2, consistent with roles in extracellular ATP clearance and ionic homeostasis [1]. Type II receptor cells detect sweet, umami, and bitter stimuli through T1R- and T2R-family receptors and the downstream signaling machinery PLCβ2, TRPM5, and α-gustducin [10]. Amiloride-sensitive sodium taste involves ENaC-expressing taste cells, but the lineage relationship of these cells to canonical type II TRCs remains incompletely resolved [1]. Type III presynaptic cells express serotonin, CAR4, synaptic proteins, and the proton channel OTOP1, and are central to sour taste transmission [11]. Basal/perigemmal progenitor cells—located outside the taste bud, around its base and lateral walls (extragemmal; Figure 1B)—express SOX2, p63, KRT5, and KRT14 and comprise heterogeneous progenitor and post-mitotic precursor states [12]. They are extragemmal, not intragemmal; this anatomical point is emphasized because depicting basal progenitors inside the bud, as in some earlier schematics, conflates their location with that of the mature cell types they generate. This cellular diversity requires continuous lineage control rather than a single terminal differentiation event. New TRCs must be specified in appropriate proportions while older cells are removed by apoptosis. The regulatory network must therefore coordinate progenitor maintenance, entry into differentiation, and cell-type allocation under both homeostatic and injury conditions.

### 2.3. Animal Models and Experimental Systems

The mouse remains the principal model for taste bud biology because of the availability of Cre and CreERT2 driver lines, including K14-Cre, K5-Cre, *Shh*-Cre, *Sox2*-CreERT2, *Lgr5*-CreERT2, and related reporters [12,13]. Tamoxifen-inducible systems permit temporal control of cell labeling and gene deletion, enabling lineage tracing of progenitor populations during homeostasis and after injury. Other vertebrate models address questions that are difficult to study in mice. Zebrafish (*Danio rerio*) provide optical accessibility and rapid genetic manipulation, although their taste buds are pharyngeal/oral rather than lingual [14]. Cichlid fish provide natural genetic variation in jaw morphology, tooth number, and taste bud density [15]. Elasmobranchs such as the catshark (*Scyliorhinus canicula*) are informative for evolutionary questions concerning shared oral epithelial progenitors and the relationship between taste and dental regenerative programs [16]. Organoid platforms are now central for mechanistic work and are discussed in Section 7.4; comparative vertebrate models are discussed in Section 7.1, Section 7.2 and Section 7.3.

## 3. Embryonic Development of Taste Papillae

### 3.1. Early Placode Formation and Papilla Morphogenesis

Taste bud development begins with thickened epithelial placodes at stereotyped positions on the dorsal tongue and along the developing CVP furrow [17]. In mice, morphological signs of FuP placodes appear around embryonic day (E)12–E13, before the onset of overt TRC differentiation at approximately E17–E18 [3]. These early placodes express *Shh*, which serves as a spatial marker of emerging gustatory epithelium and contributes to papilla patterning. This tissue-level patterning role is distinct from—and should not be conflated with—the later cell-level effects of HH signaling on taste cell differentiation before and after birth (Section 3.5). Jung and colleagues mapped the expression of *Shh*, *Bmp2*, *Bmp4*, and *Fgf8* during early tongue papilla development [18]. *Shh* expression was strongest in placode cores, *Bmp4* was enriched near placode edges, and *Fgf8* was detected in inter-papillary epithelium. These spatial relationships support a reaction–diffusion-like patterning model in which local placodal signals and surrounding modulatory inputs jointly determine papilla spacing [17,18].

Whether SHH signaling is functionally necessary for this patterning was addressed by subsequent studies. Work from Hall et al. provided the key gain-of-function evidence. They demonstrated that applying exogenous SHH protein or pharmacologically activating the HH pathway disrupted the normal array of FuP, leading to papilla loss, fusions, and incorrect positioning [19]. These data are most parsimoniously interpreted as demonstrating that SHH activity, when present at non-physiological levels, is sufficient to perturb the patterning field; they do not, on their own, establish that endogenous SHH is the single primary patterning cue. Later work from Liu and colleagues extended this view by identifying multiple HH signaling centers—including one in the mesenchyme—that work together with epithelial SHH to control not only the initial formation of papillae but also the maintenance of taste buds later on [20].

It is now known that the *Shh*-expressing cells of the placode are direct precursors to mature TRCs. Lineage tracing experiments using an *Shh*-Cre driver have shown that these *Shh*-positive placode cells give rise to type I and type II taste cells in the developing bud, establishing this embryonic placode as a major developmental origin of taste receptor cells. Whether type III cells also derive from these *Shh*^+^ placode progenitors remains an open question; current *Shh*-Cre lineage data are most strongly supportive for type I and type II origins [13].

### 3.2. WNT–SHH Crosstalk in Embryonic Papilla Patterning

**Figure 2:**
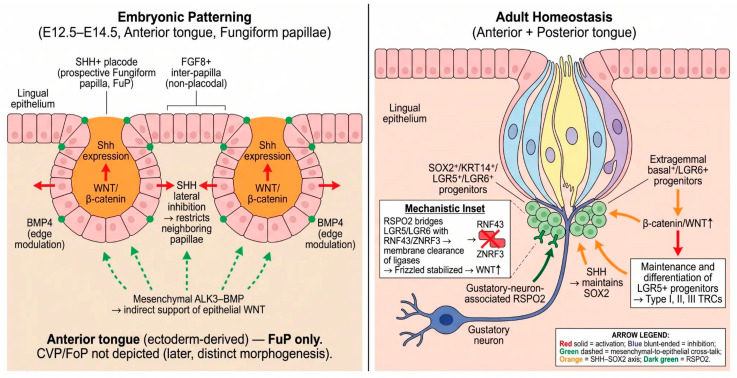
Core genetic network governing embryonic papilla patterning (**left**) and adult homeostasis (**right**). (**left**) Embryonic patterning (E12.5–E14.5, anterior tongue, fungiform papillae). WNT/β-catenin signaling activates *Shh* expression in SHH^+^ placodes (prospective fungiform papillae). SHH exerts lateral inhibition on neighboring cells, restricting adjacent placode formation and establishing the periodic FuP pattern. FGF8^+^ inter-papilla zones mark non-placodal epithelium. BMP4 modulates placode edges, and mesenchymal ALK3–BMP signaling (green dashed arrows) indirectly supports epithelial WNT activity. (**right**) Adult homeostasis (anterior + posterior tongue). Gustatory neurons supply SHH (orange arrows), which maintains SOX2 expression in progenitors. Neuronal RSPO2 (dark green arrow) amplifies WNT signaling through LGR5/LGR6 receptors by bridging them with RNF43/ZNRF3, inducing membrane clearance of the ligases, and thereby stabilizing Frizzled at the cell surface. β-catenin/WNT activity drives differentiation of LGR5^+^ progenitors into mature type I, II, and III TRCs. Mechanistic inset shows the RSPO2–LGR5/LGR6–RNF43/ZNRF3 interaction. Arrow legend: red solid = activation; blue blunt-ended = inhibition; green dashed = mesenchymal-to-epithelial cross-talk; orange = SHH–SOX2 axis; dark green = RSPO2.

Fungiform papilla patterning depends on crosstalk between WNT/β-catenin and SHH signaling (Figure 2). Iwatsuki et al. reported that epithelial β-catenin activity is required upstream for *Shh* expression in developing placodes; loss of β-catenin abolished placodal *Shh* expression and impaired papilla formation, whereas β-catenin gain-of-function expanded the placode domain [21]. Thus, WNT/β-catenin activity promotes placode formation and placodal *Shh* expression. SHH signaling then refines papilla number and spacing and restricts papilla formation in adjacent epithelium. This should not be simplified as a high-SHH inter-papillary state, because *Shh* itself is a placodal core marker. The WNT–SHH module is modulated by additional pathways. BMP signaling helps limit or refine placodal patterning, and *Fgf8* expression in inter-papillary epithelium marks or modulates non-placodal regions [18]. Mesenchymal inputs, discussed below, further influence epithelial WNT activity and differentiation competence. Papilla spacing is therefore best described as an integrated field effect involving WNT-driven placode competence, placodal *Shh* expression, and BMP/FGF-mediated modulation, rather than as a one-directional SHH-to-FGF8 cascade. On a comparative note: zebrafish data (Section 7.1) and cichlid data (Section 7.2) indicate that the same WNT–SHH–BMP toolkit is reused in non-mammalian vertebrates, but with species-specific quantitative biases; for example, in cichlids, shared loci control both tooth and taste bud density, suggesting that WNT/BMP set-points are tuned downstream of a conserved patterning logic [15].

### 3.3. SOX2 as a Competence Factor for Taste Fate

The HMG-box transcription factor SOX2 has a required role in taste lineage competence. *Sox2* hypomorphic mice form papillae but fail to generate normal taste bud sensory cells, indicating that SOX2 is not required for initial papilla morphogenesis but is essential for subsequent sensory cell differentiation [22]. This phenotype separates papilla formation, which depends heavily on WNT/SHH patterning, from taste bud cell specification, which requires SOX2-dependent competence. In the adult, high SOX2 protein levels are observed in basal epithelial cells surrounding and underlying taste buds, whereas lower levels occur in differentiating taste cells [23]. Shechtman et al. found that SOX2-high posterior lingual progenitors efficiently generate taste organoids in vitro, whereas SOX2-medium and SOX2-low populations have reduced or absent taste lineage capacity [24]. Such results support a model in which SOX2 helps maintain a taste-permissive progenitor state. We emphasize that direct genome-wide SOX2 binding targets in native taste progenitors are not yet defined; SOX2-dependent transcriptional models proposed in the literature (including downstream effects on *Pou2f3*, *Nkx2-2*, or *Notch* loci) therefore remain hypotheses awaiting ChIP-seq, CUT&RUN, or CUT&TAG validation in sorted taste progenitors. We use ‘SOX2-dependent competence’ as a functional description, not as a claim of direct chromatin occupancy.

### 3.4. Mesenchymal Control of Epithelial Taste Patterning

Taste papilla development is not purely epithelial-autonomous. Liu et al. demonstrated that neural crest-derived mesenchyme contributes to lingual mesenchyme and is required for normal papilla positioning and development [25]. Ishan et al. further demonstrated that conditional deletion of the BMP type I receptor ALK3 in tongue mesenchyme disrupts epithelial WNT/β-catenin activity and taste papilla cell differentiation [26]. These data indicate that mesenchymal ALK3–BMP signaling indirectly supports epithelial WNT activity and papilla differentiation, although the specific downstream secreted factors remain to be fully resolved. The mesenchymal–epithelial axis adds an important interpretive layer: taste bud formation and maintenance involve communication between neural crest-derived mesenchyme and epithelium, and perturbing one compartment can alter signaling in the other. This is particularly relevant when interpreting differences between anterior (ectoderm-derived) and posterior (endoderm-derived) taste fields, because the embryonic mesenchymal environments of these two territories are themselves distinct.

Critical synthesis (BMP/FGF/mesenchyme) is as follows. The available evidence for a BMP/FGF-mesenchymal contribution to taste patterning rests on a small number of conditional deletion studies, predominantly in mouse FuP [18,25,26], and the comparable experiments in posterior CVP/FoP mesenchyme have not been performed. Organoid systems (Section 7.4) recapitulate epithelial-autonomous responses to BMP and FGF ligands but lack neural-crest-derived mesenchyme, so they cannot adjudicate which mesenchymal–epithelial interactions are causal in vivo. Comparative non-mammalian data are even sparser: cichlid QTL studies implicate BMP loci in taste/tooth density coregulation [15] but do not resolve a cell-type-specific source. We therefore frame BMP/FGF/mesenchymal regulation as a confirmed but incompletely mapped layer, in which mouse anterior FuP data should not be uncritically generalized to posterior CVP/FoP or to non-mammalian models.

### 3.5. Birth as the Developmental Switch Point

A major transition in taste bud biology occurs around birth. Golden and colleagues demonstrated that taste bud cell renewal begins at or shortly after birth and coincides with a functional shift in SHH function [27]. Before birth, HH signaling in forming taste bud placodes can repress premature taste cell differentiation, despite its role in patterning taste organs. After birth, SHH becomes pro-differentiation and supports ongoing production of new TRCs from progenitors [27]. The molecular basis for this switch is not fully resolved. One plausible mechanism involves a change in the GLI transcription factor code. Available data are consistent with GLI2—dominant pre-natally—contributing to the repression of taste differentiation genes in the embryo, and GLI1 (a pure activator) becoming relatively more dominant in postnatal progenitors and promoting taste cell specification. We present this as a working model rather than an established mechanism. Golden et al. went on to identify *Foxa1* and *Foxa2* as candidate downstream targets of the postnatal SHH signal, suggesting that these Forkhead-domain transcription factors relay the pro-taste SHH output in the neonatal progenitor compartment [27]. Whether this involves direct GLI binding to *Foxa1*/2 enhancers or more indirect mechanisms, however, remains an open question that chromatin-level analysis has yet to answer.

## 4. Progenitor Cells and Lineage Specification

### 4.1. KRT14/KRT5 Progenitors and the Extragemmal Origin of New Cells

New taste receptor cells arise from KRT14^+^/KRT5^+^ basal progenitors located outside the taste bud (extragemmal), as confirmed by inducible K14-CreERT2 and K5-CreERT2 lineage tracing [12,13]. These progenitors are heterogeneous and bipotent at the population level: depending on local niche signals, individual labeled cells can give rise to either taste lineage cells or non-taste keratinocytes [12]. This anatomical clarification—basal progenitors are extragemmal, not intragemmal—is reflected in the revised Figure 1B.

### 4.2. LGR5 and LGR6 Stem/Progenitor Compartments

LGR5 and LGR6 mark partially overlapping populations of WNT-responsive epithelial progenitors. In the posterior tongue, lineage tracing from *Lgr5*-CreERT2 has identified a tripotent progenitor population capable of generating taste bud cells, surrounding non-taste epithelium, and salivary gland-like cells, indicating broader developmental potential than originally attributed to these cells [28,29,30,31]. LGR6 expression predominates in anterior fungiform progenitors, where it cooperates with LGR5 to support RSPO-amplified WNT signaling [32]. *Lgr5* and *Lgr6* are best regarded as overlapping markers of a regionally biased WNT-responsive compartment rather than as labels of a single homogeneous stem cell population—a distinction relevant to the interpretation of region-specific phenotypes (Section 2.1 and Section 8.3).

### 4.3. SOX2-High Competence and Progenitor Heterogeneity

SOX2 protein levels are graded across the basal compartment, with the highest levels in cells immediately surrounding taste buds. SOX2-high cells preferentially generate taste organoids *in vitro* and are most responsive to RSPO/WNT and HH inputs [24]. SOX2 should therefore be viewed as a marker of a competence state rather than as a binary stem cell identifier; the same epithelium contains SOX2-medium and SOX2-low cells whose lineage output is biased away from the taste fate. As stated in Section 3.3, the direct chromatin targets of SOX2 in these progenitors remain to be defined.

### 4.4. Type II Lineage Specification: POU2F3 as the Primary Lineage Selector

**Figure 3:**
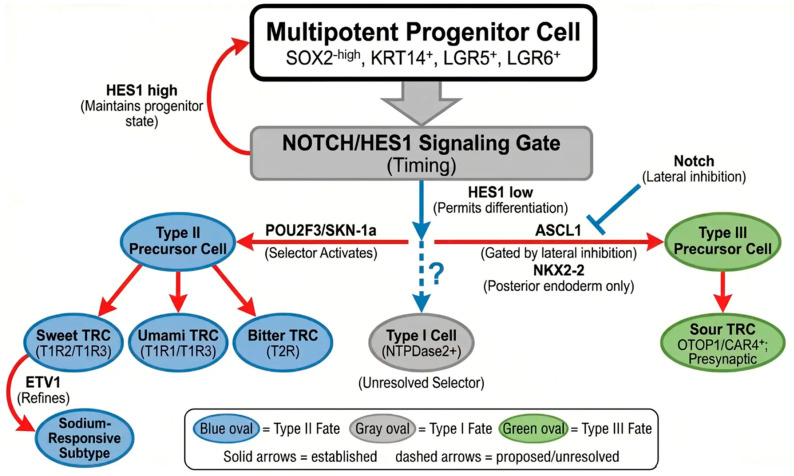
Lineage specification network within the taste bud. Multipotent progenitor cells (SOX2-high, KRT14^+^, LGR5^+^, LGR6^+^) enter a NOTCH/HES1-associated signaling gate that regulates differentiation timing. When HES1 levels are high, the progenitor state is maintained; when HES1 levels decrease, cells are permitted to differentiate. POU2F3/SKN-1a acts as the primary lineage selector for the type II receptor cell lineage, generating sweet TRCs (T1R2/T1R3), umami TRCs (T1R1/T1R3), and bitter TRCs (T2R). ETV1 further refines type II subtype programs, including sodium-responsive cells. ASCL1 promotes type III presynaptic cell differentiation, gated by Notch-mediated lateral inhibition (blue blunt-ended arrow). NKX2-2 promotes type III lineage specification, specifically in posterior endoderm-derived taste fields (CVP and FoP). Type III sour TRCs express OTOP1, CAR4^+^, and are presynaptic. The transcriptional selector for type I cells (NTPDase2^+^) remains unresolved (dashed arrow with question mark). Legend: blue ovals indicate type II fate; gray ovals indicate type I fate; green ovals indicate type III fate. Solid arrows represent established pathways; dashed arrows represent proposed or unresolved connections.

POU2F3/SKN-1a is the primary lineage selector for type II taste receptor cells (sweet, umami, and bitter; Figure 3). *Pou2f3* loss eliminates type II TRCs responsible for sweet, umami, and bitter detection, including cells expressing TRPM5 and PLCβ2 [33,34]. *Pou2f3* loss eliminates type II TRCs without obvious effects on type I or type III populations, while leaving general epithelial architecture intact, demonstrating that POU2F3 functions at the lineage allocation step rather than at the level of progenitor maintenance. POU2F3 is selectively required for type II fate specification but is insufficient to drive the program in non-taste-competent epithelium without SOX2-dependent competence, warranting the more precise term lineage selector rather than master regulator. On a comparative note: POU2F3 is reused outside taste, including in tuft cells of the gastrointestinal and respiratory mucosa, indicating that the same lineage selector has been redeployed across chemosensory epithelia.

### 4.5. Type III Lineage Specification: ASCL1, with Posterior-Field NKX2-2

Type III (sour, presynaptic) cells require ASCL1 as a lineage-restricted transcription factor [35]. In the posterior tongue, NKX2-2 is additionally required for normal type III specification, indicating that the type III program is regionally tuned and that the anterior (ectoderm-derived) and posterior (endoderm-derived) taste fields are not identical at the lineage selector level [8]. This region-specific requirement underlies our recommendation (Section 2.1) that perturbations of any single component be interpreted within the appropriate anterior vs. posterior context. The molecular target genes of ASCL1 and NKX2-2 in taste progenitors remain to be defined directly by ChIP-seq.

### 4.6. Notch/HES Signaling as a Timing and Gating Mechanism

*Notch* signaling, with HES1 as the principal effector, controls the timing and balance of taste cell differentiation [36,37]. *Notch* activation in progenitors suppresses premature differentiation, while *Notch* downregulation in committed precursors permits lineage progression. This timing/gating role complements—rather than parallels—the lineage selector roles of POU2F3 and ASCL1: *Notch* determines when a competent progenitor differentiates, whereas POU2F3/ASCL1 determines which lineage it enters.

Critical synthesis (*Notch*/HES). The *Notch* pathway is conserved across mouse, zebrafish, and organoid systems, in each case acting as a timing gate rather than as a lineage selector; this convergence is among the more robust cross-system observations in taste biology. Two caveats remain. First, the published evidence relies primarily on pharmacological γ-secretase inhibition and on *Hes1* reporter analyses; cell-type-resolved chromatin data for *Notch*-responsive enhancers in native taste progenitors are still lacking. Second, the proportional contribution of *Notch* to type II vs. type III lineage allocation, and whether posterior NKX2-2-positive progenitors use a *Notch* threshold different from anterior progenitors, have not been quantified. The current model should therefore be read as a conserved gating logic with regional parameters that remain to be measured.

### 4.7. Emerging Regulators and Unresolved Lineages

Several gaps in the lineage map remain. The selector for type I glial-like cells has not been identified; candidate factors (GFI1, SOX10, NFIA) are expressed but have not been validated by conditional knockout in taste tissue, and a default-fate model—type I identity arising in the absence of POU2F3 or ASCL1 activity—cannot be excluded. The lineage origin of amiloride-sensitive ENaC^+^ sodium-responsive cells is similarly unresolved and should not be assigned to the type II lineage by default. These open issues are revisited in the Outstanding Questions section (Section 8.3).

## 5. Adult Homeostasis and Signaling Maintenance

### 5.1. HH/GLI Signaling as a Homeostatic Requirement

SHH signaling illustrates how a developmental pathway is repurposed for adult tissue maintenance. Blocking HH signaling in adult mice, either by conditional HHIP expression or by SMO inhibition, rapidly reduces progenitor proliferation and shrinks taste cell populations [5,23]. Conversely, conditional HH activation can maintain or expand taste bud cell populations [38]. The results establish HH signaling as a continuing requirement for adult taste cell turnover, not merely an embryonic patterning cue. Adult SHH has both epithelial and neuronal sources. SHH protein is produced by basal precursor cells within taste buds and by gustatory neurons [39]. Neuron-specific or epithelium-specific *Shh* deletion alone produces modest effects on taste bud maintenance, but combined loss of both neuronal and epithelial SHH causes severe taste bud degeneration, demonstrating that SHH from both sources cooperatively maintains the taste epithelium [39]. *Shh*-expressing basal cells are post-mitotic precursors of functional TRCs, linking *Shh* expression to the differentiation trajectory of taste cells [40].

### 5.2. Downstream of HH in Adult Tissue Maintenance

HH activity is linked to SOX2-dependent progenitor maintenance. In adult lingual epithelium, SMO inhibition rapidly reduces SOX2 protein in progenitor populations, and loss of SOX2 disrupts production of differentiated taste cells [23]. Ohmoto et al. further demonstrated that *Sox2* deletion in posterior tongue epithelium disrupts both taste bud cells and LGR5^+^ progenitor identity [41]. These studies support an HH–SOX2–LGR5 regulatory axis, although the direct chromatin intermediates and GLI-dependent enhancers remain to be defined.

Critical synthesis (HH/GLI in homeostasis and across models) is as follows. Adult HH signaling is required for taste cell turnover in mouse, and the same pathway is repurposed embryonically (Section 3.5), with the GLI2-to-GLI1 transition around birth representing a plausible—but not directly demonstrated—molecular substrate of the functional switch. In organoid cultures, removal of SHH or pharmacological SMO inhibition disrupts taste cell generation, supporting an epithelium-autonomous arm of the pathway; however, organoids cannot capture the in vivo neuronal SHH source documented by Castillo-Azofeifa et al. [39]. Cross-species data are limited: zebrafish taste bud development requires Hedgehog input but the adult homeostatic equivalent is not well-characterized in fish. We therefore present HH/GLI as a conserved developmental cue with a homeostatic role established primarily in mouse, while the chromatin-level connection between GLI output and SOX2/LGR5 transcription remains an unmet experimental need.

### 5.3. LGR5/RSPO/WNT Axis and the Neural Niche

The WNT/β-catenin pathway is also indispensable for adult taste cell turnover. Conditional deletion of β-catenin in K5+ progenitors causes progressive loss of taste progenitors, collapse of SHH+ precursor populations, and impaired behavioral taste perception [42]. Thus, WNT signaling is required for progenitor maintenance and taste cell turnover. Whether WNT activity directly instructs specific mature TRC subtypes or primarily acts through progenitor competence and survival remains an area requiring further primary evidence [9]. Adult WNT tone is regulated by R-spondin proteins. RSPOs bind LGR4/5/6 co-receptors and bridge them to the transmembrane E3 ubiquitin ligases RNF43 and ZNRF3, inducing membrane clearance of the ligases. In the absence of this clearance, RNF43/ZNRF3 ubiquitinate Frizzled and promote its turnover; clearance therefore stabilizes Frizzled at the cell surface [43]. Stabilizing Frizzled receptors increases progenitor responsiveness to available WNT ligands. In the taste epithelium, epithelial deletion of *Rnf43*/*Znrf3* expands the progenitor pool and demonstrates that endogenous RSPO/RNF43/ZNRF3 regulation limits taste tissue size [43]. Lin et al. reported that exogenous RSPO1 can partially substitute for neuronal input after glossopharyngeal nerve transection and can help maintain taste cell generation in denervated animals [44]. In organoid cultures, removal of R-spondin markedly impairs generation of differentiated taste cells, reinforcing the importance of RSPO-amplified WNT signaling for epithelial progenitor function [32,44].

**Figure 4:**
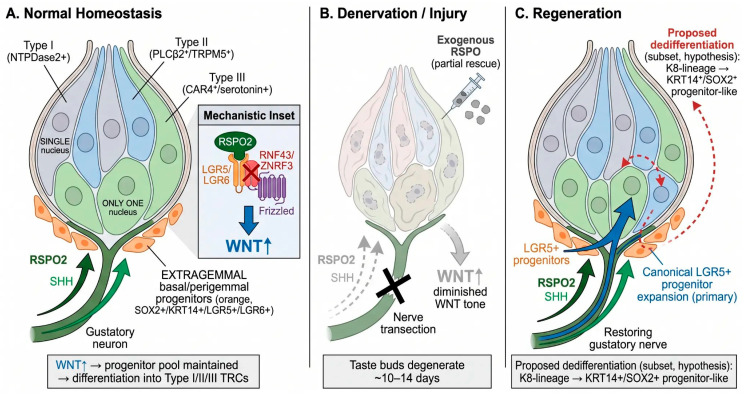
Neural niche model and regeneration mechanisms. (**A**) Normal homeostasis. Gustatory neurons supply RSPO2 (dark green arrow) and SHH (green arrow) to extragemmal basal/perigemmal progenitors (SOX2^+^/KRT14^+^/LGR5^+^/LGR6^+^). RSPO2 amplifies WNT signaling through LGR5/LGR6 receptors by bridging them with the transmembrane E3 ubiquitin ligases RNF43/ZNRF3 (red X), inducing their membrane clearance and thereby stabilizing Frizzled at the cell surface. SHH supports SOX2 expression and progenitor identity. Active WNT signaling maintains the progenitor pool and drives ongoing differentiation into type I, II, and III TRCs; each TRC is drawn with a single nucleus. (**B**) Denervation/injury. Nerve transection (black X) eliminates neuronal RSPO2 and SHH supply. Taste buds degenerate over approximately 10–14 days. Exogenous RSPO (syringe) can partially rescue taste bud integrity. (**C**) Regeneration. Reinnervation restores neuronal RSPO2 and SHH support. Regeneration proceeds primarily through canonical LGR5^+^ progenitor expansion (blue arrow). A dashed red arrow indicates a proposed, subset-level dedifferentiation pathway (hypothesis): K8-lineage cells may acquire KRT14^+^/SOX2^+^ progenitor-like features.

Xu et al. examined the requirement for *Rspo2* in vivo using a hypomorphic allele that reduces *Rspo2* expression broadly rather than specifically in gustatory neurons [6]. In that model, taste bud number was reduced in both the anterior and posterior tongue while nerve fiber anatomy remained intact; in the same study, AAV-hSyn-Cre-mediated ablation of *Rspo2* specifically in the nodose-petrosal-jugular (NPJ) ganglion complex of *Rspo2*Gem-fl/Gem-fl mice led to near-complete loss of circumvallate taste buds, with a strong correlation between residual *Rspo2* expression and remaining taste bud number (Figure 4). These findings directly demonstrate that gustatory-neuron-derived RSPO2 is required for taste bud maintenance, although a germline Cre driver specific to gustatory neurons remains unavailable. Within these limits, the data support a working model in which gustatory neurons supply RSPO2 to potentiate LGR5/LGR6-dependent WNT signaling in adjacent progenitors, with niche support directed from neuron to epithelium rather than the reverse.

Critical synthesis (WNT and RSPO niche) is as follows. The WNT requirement for adult taste bud maintenance is well-supported across mouse genetic models, organoid systems, and pharmacological rescue studies; in this respect, it is the single most robust signaling claim in the field. Where evidence remains thinner is in the cellular source of the niche RSPO2, the relative contribution of LGR5 vs. LGR6 in anterior FuP vs. posterior CVP/FoP, and the cross-species generalizability of the LGR5/LGR6–RSPO–WNT axis (current human data are limited to LGR5/LGR6 expression in CVP organoids; functional dependence on RSPO has not been formally demonstrated in human tissue). We therefore endorse the LGR5/LGR6–RSPO2–WNT axis as a well-supported mouse model of the taste bud neural niche, while noting that (i) cell-source-specific RSPO2 confirmation, (ii) regional LGR5/LGR6 dissection, and (iii) human validation are each open experimental needs.

## 6. Injury and Regeneration

### 6.1. Denervation as the Canonical Regeneration Model

Surgical transection of the chorda tympani or glossopharyngeal nerves is the canonical injury model in this field. Following nerve injury, taste buds in the deafferented field degenerate over approximately 10–14 days, with loss of mature type I/II/III TRCs and shrinkage of the bud (Figure 4B). Reinnervation, when permitted, restores epithelial RSPO2 and SHH input from regenerating fibers and is followed by regeneration of taste buds (Figure 4C). *Lgr5*-CreERT2 lineage tracing has confirmed that canonical LGR5^+^ progenitor expansion accounts for the bulk of regenerated TRCs after reinnervation; exogenous RSPO can partially preserve taste bud cell number in denervated animals, consistent with the niche role of neuronal RSPO2 [6,44].

### 6.2. Adult Epithelial Competence Revealed byShhMisexpression

Adult lingual epithelium retains a latent capacity to form taste-bud-like structures, as revealed by forced epithelial *Shh* expression, which can induce ectopic taste-bud-like cell clusters outside normal papilla territories. The competence to form a bud is therefore not strictly limited to embryonic placodes. However, the persistence of these ectopic buds requires continued neural input, again pointing to a neuron-to-epithelium niche dependence. We frame this as evidence that adult epithelial competence is broader than its normal spatial deployment, not as evidence that any region of the adult tongue is an equivalent substrate for taste bud formation.

### 6.3. Radiation-Induced Injury and WNT-Mediated Rescue

Ionizing radiation causes taste dysfunction in part by depleting cycling KRT14^+^/LGR5^+^ progenitors through cell-cycle arrest and apoptosis. In mouse models, WNT-stabilizing interventions—for example, GSK3β inhibition by lithium chloride—partially mitigate progenitor loss and accelerate recovery of taste epithelium [45,46,47]. The same studies make clear that such recovery is dependent on a residual progenitor pool; pharmacological WNT amplification cannot regenerate taste buds in epithelium that has been fully ablated. As discussed in Section 8.2, this places stringent constraints on translational deployment of WNT amplifiers in patients with active or recently treated malignancies of the head and neck.

### 6.4. Injury-Induced Epithelial Plasticity and Dedifferentiation

A subset of K8-lineage differentiated taste receptor cells can acquire KRT14^+^/SOX2^+^/PCNA^+^ progenitor-like features under defined injury conditions, suggesting a context-restricted dedifferentiation pathway distinct from canonical LGR5^+^ progenitor expansion [48]. This should be regarded as a supplementary, hypothesis-level pathway rather than a primary mode of regeneration: the proportion of regenerated TRCs derived from dedifferentiation has not been quantified at the population level, the injury contexts in which it occurs have not been systematically compared (denervation vs. irradiation vs. chemical injury), and the molecular signals that license dedifferentiation remain undefined. Whether this process contributes to normal homeostatic turnover or represents a strictly stress-induced response is an open question.

## 7. Comparative and Emerging Perspectives

### 7.1. Zebrafish: Mechanistic Genetics of Taste Bud Ontogeny

Zebrafish (*Danio rerio*) provide a tractable system for in vivo genetic manipulation of taste bud development [49]. Studies in zebrafish have established that Hedgehog, *Notch*, and FGF signaling cooperate to specify oral taste bud progenitors, and that FGF inputs converge onto *Notch*-dependent lineage gating in a manner broadly analogous to mouse *Notch*/HES timing logic. We note two limitations relevant to direct extrapolation: zebrafish taste buds are oral/pharyngeal rather than lingual, and the morphogenetic context (no papilla per se) differs substantially from mammalian fungiform/circumvallate organization. The comparative value of zebrafish is therefore strongest at the level of conserved pathway logic (e.g., *Notch* as a timing gate), and weakest at the level of papilla-specific patterning.

### 7.2. Cichlid Fish: Coevolution of Taste and Dental Structures

Cichlid fish provide natural genetic variation in jaw morphology, tooth number, and taste bud density. Quantitative trait locus mapping shows that overlapping genomic regions control tooth and taste bud density, and that this coregulation is largely encoded in the WNT, BMP, and Hedgehog toolkits. This is consistent with the view that vertebrate oral epithelium uses a shared progenitor competence platform that can be channeled toward dental or gustatory lineages depending on local signaling. The relevance to mammalian biology is conceptual rather than mechanistic, and supports the broader argument that taste epithelial progenitors share evolutionary roots with other oral epithelial progenitors.

### 7.3. Sharks: Evolutionary Origin of the SOX2^+^ Oral Progenitor

Catshark (*Scyliorhinus canicula*) and related elasmobranchs are informative for tracing the evolutionary origin of SOX2^+^ oral progenitors. SOX2^+^ progenitor cells in shark oral epithelium contribute both to taste buds and to continuous tooth replacement, indicating that the SOX2^+^ oral progenitor predates the cartilaginous-vs.-bony fish divergence and is conserved across jawed vertebrates [16]. We frame this as evidence of a conserved cell type, not as evidence of identical regulatory networks: the downstream lineage selectors POU2F3, ASCL1, and NKX2-2 have not been systematically interrogated in elasmobranchs, and the taxonomic sampling underlying current evolutionary inferences remains sparse.

### 7.4. Organoid Platforms: Bridging Genetics and Physiology

Taste organoids can be established from single LGR5+ or LGR6+ progenitors and reconstitute multiple TRC lineages under defined culture conditions [32,50]. Recent refinements include surface-accessible organoid formats that permit electrophysiological recording, organoids with improved functional maturation of type II transduction machinery, and region-specific anterior tongue organoids that allow direct comparison with posterior CVP-derived organoids [51,52]. Because most current systems lack gustatory neurons, mesenchyme, and vascular/immune inputs, organoids are best regarded as reductionist models of epithelial competence and lineage allocation, not as full replicas of the *in vivo* taste bud niche. We use organoid evidence in this review when it directly constrains epithelial-autonomous mechanisms (e.g., the requirement for R-spondins in TRC generation; Section 5.3) and avoid using it to infer in vivo neural niche function.

### 7.5. Single-Cell Genomics and Atlas Approaches

Single-cell RNA sequencing has resolved progenitor and TRC heterogeneity in mouse taste papillae, defined aging signatures in a CVP single-cell atlas, and identified a tripotent posterior LGR5^+^ population capable of generating taste, non-taste epithelial, and salivary gland lineages. We highlight this not as a redefinition of LGR5 as a ‘super-stem’ marker, but as evidence that lineage potential within the LGR5^+^ compartment is broader and more regionally biased than originally proposed. Critical chromatin-accessibility comparisons (scATAC-seq) between anterior LGR6^+^ and posterior LGR5^+^ progenitors are needed to determine whether the anterior–posterior network differences identified at the lineage selector level (Section 2.1 and Section 4.5) are reflected in differential enhancer usage.

### 7.6. Metabolic Disease, Viral Infection, and Emerging Modulators of Taste Homeostasis

Metabolic disorders are increasingly recognized as contributors to taste dysfunction. Kaufman et al. reported that obesity-associated low-grade inflammation reduces taste bud abundance in mice, linking this effect to TNF-α-mediated suppression of progenitor renewal [53]. GLP-1-related pathways may also influence taste receptor expression, taste sensitivity, and food preference, although the direct effects of GLP-1 receptor agonists on taste bud lineage networks require further mechanistic investigation [54]. COVID-19 has drawn clinical attention to persistent taste dysfunction. Yao et al. reported long-term morphological changes in taste papillae following SARS-CoV-2 infection [55], and Morad et al. identified reduced PLCβ2 and TAS1R3 expression in taste tissues from individuals with long COVID taste dysfunction [56], suggesting that persistent taste loss may involve impaired maintenance or function of PLCβ2-dependent type II taste pathways. The cellular site of SARS-CoV-2 entry in the lingual epithelium remains unsettled. Some studies report ACE2 expression in type II taste receptor cells with evidence of direct infection ([55] and references therein), whereas others report ACE2 enrichment in basal cells of non-gustatory papillae and in keratinocyte subpopulations rather than within taste buds. Whether persistent taste dysfunction reflects direct infection of taste cells, indirect effects on the supporting epithelium, or post-infectious inflammation that perturbs POU2F3-dependent lineage specification, WNT/*Notch* signaling, or neuronal RSPO2 support remains to be resolved.

Complementing single-cell sequencing, spatial transcriptomics now allows resolution of gene expression patterns within the taste organ native environment. Using tomo-seq on the mouse oral mucosa—spanning the tongue; cheeks; and palate—Seubert et al. identified FGF pathway components such as FGF1; FGF7; and FGF10 as site-specific niche factors [57]. Organoid assays confirmed their importance. Spatial mapping is crucial for addressing the fundamental question of why taste buds form only in specific locations and how their local signaling microenvironment is established.

An unanticipated regulatory layer has emerged from the intersection of chronobiology and single-cell genomics. Matsu-Ura et al. discovered that circadian clock genes are expressed heterogeneously across taste bud cells [58]. Clock-gated cell renewal produces time-of-day-dependent changes in taste sensitivity in mice. Their scRNA-seq data revealed that gene expression in type II taste cells varies diurnally, suggesting the entire output of the taste bud genetic network is temporally modulated. Such observations have immediate implications for experimental design, as time-of-day effects must now be considered. They also provide a potential mechanism explaining how aging, known to disrupt circadian rhythms, might exacerbate the age-related decline in taste progenitor function documented by Ren et al. [59].

## 8. Translational Implications

### 8.1. Taste Dysfunction: Clinical Context and Mechanistic Framework

Clinically, taste dysfunction—whether as distorted taste (dysgeusia) or its complete loss (ageusia)—is a significant problem with a wide array of causes. The etiologies range from head and neck radiation therapy and chemotherapy to viral infections such as SARS-CoV-2, aging, and zinc deficiency. Many medications also contribute; for instance, the Hedgehog pathway inhibitors used to treat basal cell carcinoma and some leukemias are well-documented causes [4]. The mouse-derived molecular framework summarized above suggests candidate mechanisms for several clinical taste disorders, which we present as hypotheses awaiting human validation rather than as established human mechanisms. Radiation-induced dysgeusia likely reflects progenitor depletion and reduced WNT/β-catenin signaling [45,46]. Chemotherapy may produce overlapping progenitor and inflammatory effects, though mechanisms likely vary by agent. In COVID-19-associated taste dysfunction, available data support altered taste bud architecture and reduced expression of type II transduction genes in some patients [55,56,60]. A contribution from gustatory neuron dysfunction or reduced RSPO2 niche support is plausible but unproven and should be presented as a hypothesis rather than an established mechanism [6]. Age-related taste decline may involve reduced epithelial renewal, altered WNT target expression, and changes in neural support, but causal relationships remain to be defined [59].

### 8.2. Pharmacological Targets for Taste Restoration

Several pharmacological targets emerge from this network. WNT amplification is the most developed conceptually: LiCl promotes recovery in irradiated mouse models, and exogenous RSPO can partially maintain taste buds after denervation [44,47]. However, systemic WNT activation has safety limitations, including proliferative and oncogenic risks. This concern is particularly relevant for head and neck cancer patients who have undergone radiation therapy: residual tumor cells in the irradiated field could be stimulated by exogenous WNT activation, potentially promoting cancer recurrence or second primary tumor development. The oral mucosa is already a site of frequent WNT pathway dysregulation in squamous cell carcinoma. Translation will therefore require local rather than systemic delivery, transient rather than sustained activation, stringent tumor surveillance protocols, and exclusion of patients with active or recently treated malignancies until adequate preclinical safety data are available. SIRT1 inhibition represents another preclinical approach for radiation-associated progenitor protection [61]. HH signaling is a double-edged target. Adult HH activity is required for taste bud homeostasis, and cancer-directed HH pathway inhibitors can disrupt taste organs [5,23]. Conversely, uncontrolled HH activation is not a straightforward therapeutic solution. Strategies that preserve gustatory neuronal support may be attractive because neurons supply both RSPO2 and SHH, but neurotrophic-factor approaches require evidence that they maintain relevant ligand secretion and translate safely to humans [39,62].

### 8.3. Outstanding Questions

A major unresolved issue is the precise difference between the gene regulatory networks governing the anterior, ectoderm-derived taste field versus the posterior, endoderm-derived one. NKX2-2 data [8] and the clear distinction between anterior and posterior organoids [52] indicate that these networks are not identical. However, a direct head-to-head genomic comparison at the chromatin level—a critical experiment—has not yet been performed. This leaves a fundamental gap in the current understanding of taste field patterning.

The identity of the transcriptional selector for type I glial-like taste cells remains unresolved. Candidate transcription factors include GFI1, which specifies secretory versus absorptive fates in intestinal epithelium and is expressed in some taste bud cells, and glia-associated factors such as SOX10 and NFIA, given the glial-like wrapping morphology and NTPDase2 expression of type I cells. However, none of these candidates has been validated by conditional knockout in taste tissue, and the possibility that type I identity arises by default in the absence of POU2F3 or ASCL1 activity—rather than through a dedicated selector—cannot be excluded. The specification of amiloride-sensitive ENaC^+^ sodium-responsive cells is also uncertain. These cells should not be described as definitively canonical type II cells until lineage, transcriptional, and functional evidence resolves whether they follow the POU2F3 program or a distinct pathway.

Although SOX2, GLI factors, β-catenin/TCF, POU2F3, NKX2-2, ASCL1, and HES1 have been implicated in taste bud biology, their direct chromatin targets and co-factor interactions in native taste progenitors and differentiating cells remain poorly defined. CUT&RUN, CUT&TAG, and chromatin-accessibility profiling of sorted cells from fresh tissue and organoids should help address this gap. In a related context, single-cell transcriptomics has shown that a subset of murine esophageal progenitors can differentiate into taste-bud-like cells *in vivo*, consistent with the broader plasticity of upper gastrointestinal epithelium and the relevance of single-cell approaches [63]. An additional area worthy of investigation concerns the role of dedifferentiation in taste bud maintenance. The extent to which the reversion of mature TRCs to a progenitor-like state contributes to regeneration following various injury types, as well as the molecular signals that enable or inhibit this lineage reversal, remains to be determined. Whether dedifferentiation constitutes a significant component of normal homeostatic turnover or is strictly a stress response remains unclear.

Perhaps the most pressing question is whether the mouse-derived networks are conserved in humans. This issue is crucial for translating mechanistic insights into human therapies. However, systematic comparative genomics between mouse and human taste cells, combined with functional validation in human organoids or non-human primate tissue, remains largely incomplete. Specific high-priority experiments include: (i) POU2F3 CUT&RUN in FACS-sorted TRPM5^+^ type II precursors to define direct versus indirect transcriptional targets; (ii) ATAC-seq comparison of anterior LGR6^+^ versus posterior LGR5^+^ progenitors to identify region-specific chromatin-accessibility landscapes; and (iii) single-cell multi-omic profiling (scRNA-seq + scATAC-seq) of taste buds during the first 72 h after denervation to capture the earliest molecular events in progenitor activation and potential dedifferentiation. Current human taste data remain sparse. The GTEx consortium provides bulk RNA-seq from human tongue tissue but lacks taste-bud-specific resolution. No dedicated human taste bud single-cell atlas comparable to the mouse CVP atlas [59] has been published to date. Preliminary evidence from human circumvallate organoids suggests that LGR5 and LGR6 are expressed in human taste progenitors, but whether their relative contributions and regional distributions mirror the mouse pattern is unknown. Human taste bud turnover has been estimated at approximately 10–14 days based on histological observations, broadly similar to mouse FuP kinetics, but systematic measurement using modern pulse-chase approaches has not been performed. Bridging this species gap is essential: generating a Human Cell Atlas-quality single-cell reference for human taste tissue, combined with cross-species computational integration, should be a priority for the field.

## 9. Conclusions

**Figure 5:**
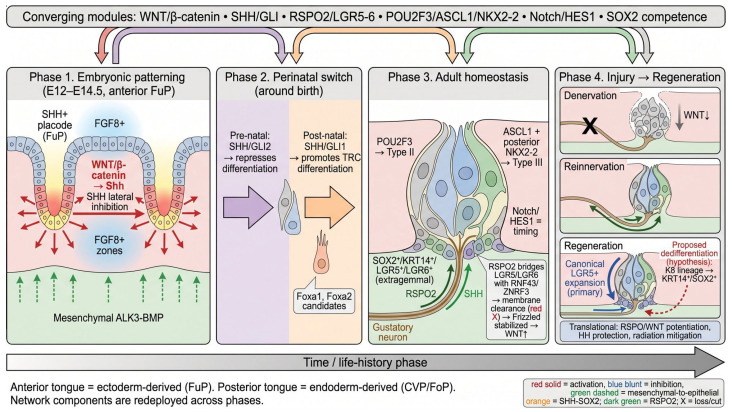
Integrated temporal map of the taste bud signaling network. A single horizontal time axis is divided into four sequential phases: (1) Embryonic patterning (E12–E14.5, anterior FuP): WNT/β-catenin drives placode formation and *Shh* expression; SHH lateral inhibition establishes the periodic pattern; mesenchymal ALK3–BMP supports epithelial WNT; Foxa1/Foxa2 are candidate endoderm regulators. (2) Perinatal switch (around birth): SHH/GLI signaling transitions from repressing differentiation (pre-natal, GLI2-mediated) to promoting TRC differentiation (postnatal, GLI1-mediated). (3) Adult homeostasis: SOX2^+^/KRT14^+^/LGR5^+^/LGR6^+^ extragemmal progenitors are maintained by neuronal RSPO2 and SHH; POU2F3 specifies type II, ASCL1 + NKX2-2 specify type III; Notch/HES1 gates differentiation timing. (4) Injury → regeneration: denervation causes WNT loss and taste bud degeneration; reinnervation restores RSPO2/SHH, enabling canonical LGR5^+^ expansion and proposed dedifferentiation (hypothesis). Converging signaling modules (top banner) are redeployed across all phases. Bottom note: anterior tongue = ectoderm-derived (FuP); posterior tongue = endoderm-derived (CVP/FoP). Arrow legend: red solid = activation; blue blunt = inhibition; green dashed = mesenchymal-to-epithelial; orange = SHH–SOX2; dark green = RSPO2; X = loss/cut.

Taste bud development, homeostasis, and regeneration are often treated as distinct biological processes; however, the evidence reviewed here indicates that they share overlapping signaling modules (Figure 5). SHH contributes first to embryonic placode patterning, shifts its functional output around birth, and subsequently supports adult progenitor maintenance. WNT/β-catenin signaling drives embryonic placode formation and sustains adult epithelial renewal, with RSPO2 from gustatory neurons serving as a key WNT-amplifying niche signal. Downstream, POU2F3/SKN-1a and ASCL1/NKX2-2-associated programs specify type II and type III lineages, respectively, while *Notch*/HES signaling regulates the timing and balance of differentiation. Together, these findings support a tissue-ecosystem model in which taste bud maintenance depends on epithelial progenitors, neural niche signals, mesenchymal inputs, and lineage-specific transcriptional programs.

Regeneration after injury involves canonical LGR5^+^ progenitor expansion and, in specific injury contexts, may be supplemented by dedifferentiation of a subset of committed TRCs, though this remains a hypothesis-level pathway whose relative contribution across injury types has yet to be quantified in vivo. The finding that c-*Kit*-expressing sweet taste cells resist nerve injury and support subsequent regeneration [64], together with evidence that tyrosine kinase inhibitors dysregulate fate selection of specific taste bud cell subtypes through KIT inhibition [65], points to an additional layer of lineage-specific resilience. From a translational standpoint, RSPO/WNT potentiation, protection of HH-dependent progenitor maintenance, mitigation of radiation-induced progenitor injury, and controlled modulation of injury-associated plasticity represent plausible therapeutic directions. Each requires careful validation, as the same pathways that promote epithelial renewal can drive pathological proliferation when activated broadly or chronically. Figure 5 summarizes this integrated network along a temporal axis, illustrating how embryonic patterning signals transition into adult homeostatic mechanisms and how injury disrupts—and reinnervation restores—these circuits. Resolving these gaps will require integration of single-cell genomics, spatial profiling, organoid systems, and validation in human or non-human primate tissue.

## Data Availability

This is a review article; no original data were generated. All data supporting the findings of this review are available within the cited references.
